# Artificial Intelligence in Cardiovascular Ultrasound: Clinical Applications, Foundation Models, and the Path to Precision Cardiology

**DOI:** 10.3390/jcm15166398

**Published:** 2026-08-19

**Authors:** Ancuta Elena Tupu, Simona Steliana Tudor, Caterina Nela Dumitru, Claudia Simona Stefan, Ionela Daniela Ferțu

**Affiliations:** 1 Faculty of Medicine and Pharmacy, Medical-Pharmaceutical Research Center, “Dunarea de Jos” University of Galati, 800008 Galati, Romania; anca.tupu@ugal.ro (A.E.T.); claudia.stefan@ugal.ro (C.S.S.); ionela.fertu@ugal.ro (I.D.F.); 2 Clinical Hospital of Infectious Diseases “St. Venerable Parascheva”, 800179 Galați, Romania

**Keywords:** artificial intelligence, machine learning, deep learning, foundation models, vision–language models, echocardiography, cardiovascular ultrasound, speckle-tracking echocardiography, global longitudinal strain, cardiovascular risk stratification, precision medicine

## Abstract

Cardiovascular ultrasound is a cornerstone of noninvasive cardiac and vascular assessment, yet conventional interpretation remains operator-dependent, variable, and limited in sensitivity for subclinical disease. Artificial intelligence (AI), particularly machine learning (ML), deep learning (DL), and, most recently, vision–language and foundation models, offers tools to automate, standardize, and extend ultrasound analysis. This narrative review examines the role of AI-enhanced cardiovascular ultrasound in the transition from descriptive imaging toward predictive and personalized medicine. We conducted a structured literature search of PubMed/MEDLINE, Scopus, Web of Science, and Google Scholar (January 2016–May 2026), combining Medical Subject Headings and free-text terms related to AI and cardiovascular ultrasound. Original studies, meta-analyses, reviews, consensus documents, and seminal works were considered. AI now spans the entire echocardiographic workflow, acquisition guidance, view classification, segmentation (Dice ≈ 0.92–0.94 on public datasets), and automated quantification of ejection fraction and global longitudinal strain, achieving expert-level accuracy and improved reproducibility. Across clinical domains, AI supports ischemia detection on stress echocardiography, heart-failure phenogrouping, Doppler-independent aortic stenosis detection, and carotid plaque characterization for stroke-risk stratification. Emerging vision–language and multitask foundation models (e.g., EchoCLIP, EchoPrime, and PanEcho) point toward general-purpose interpretation, and a growing number of tools (Caption Guidance, Us2.ai, and Ultromics EchoGo) have obtained FDA clearance and/or CE marking. Increasingly, AI-derived imaging biomarkers feed multimodal models that enable individualized risk prediction and therapy selection. AI-enhanced cardiovascular ultrasound is poised to become a central tool of precision cardiology. Realizing its potential will require prospective multicenter validation, cross-vendor standardization, attention to generalizability, interpretability, and reproducibility, and evolving regulatory and ethical frameworks.

## 1. Introduction

Cardiovascular diseases (CVDs) remain the leading cause of morbidity and mortality worldwide, accounting for approximately 19 million deaths in 2020 and imposing a substantial socioeconomic burden on health-care systems [1]. The rising prevalence of cardiovascular risk factors, including arterial hypertension, diabetes mellitus, obesity, and population aging, has created a growing need for diagnostic methods capable of detecting cardiovascular impairment early and enabling personalized therapeutic strategies. Cardiovascular imaging has consequently become a cornerstone of modern medicine, contributing to early diagnosis, prognostic assessment, and monitoring of disease progression.

Cardiovascular ultrasound occupies a central role in the noninvasive evaluation of the heart and vascular system owing to its accessibility, nonionizing nature, relatively low cost, and ability to provide morphologic and hemodynamic information in real time. Over recent decades, ultrasound techniques have evolved from purely anatomic assessment toward functional and biomechanical characterization of cardiovascular structures, aided by speckle-tracking echocardiography (STE), three-dimensional echocardiography, and contrast-enhanced ultrasound [2,3]. Nevertheless, conventional interpretation remains largely dependent on operator experience, with substantial inter- and intra-observer variability [4]; subclinical impairment is difficult to capture with traditional parameters; and the growing complexity of imaging data increasingly exceeds the capacity of subjective visual assessment [5].

Against this background, artificial intelligence (AI) has opened new perspectives in medical imaging. Machine learning (ML) and its subset deep learning (DL) learn complex patterns directly from data and, applied to ultrasound, enable automated acquisition, segmentation, functional quantification, and predictive modeling [6,7,8,9]. Most recently, the field has moved beyond narrow, single-task convolutional networks toward transformer-based, self-supervised, and vision–language foundation models trained on millions of studies, alongside large language models (LLMs) for reporting and clinical decision support, developments that are reshaping expectations of what AI-enabled echocardiography can deliver. Integrating AI into cardiovascular ultrasound reduces operator-dependent variability, improves reproducibility, and enables earlier identification of subtle abnormalities that may precede overt disease [4,10]. This transformation is closely tied to predictive and personalized medicine, in which therapeutic decisions are tailored to the individual patient through the integration of imaging, clinical, and biological data [11,12], and it may extend cardiovascular screening and point-of-care ultrasound (POCUS) to resource-limited settings and telemedicine platforms [13,14,15].

This narrative review critically examines the role of AI integrated with advanced cardiovascular ultrasound in transforming diagnosis, prognostic assessment, and personalized medicine. We evaluate the main clinical applications, emerging architectures and imaging biomarkers, the regulatory landscape of approved tools, and the current advantages and limitations of AI technologies, with attention throughout to quantitative performance, generalizability, and reproducibility.

A narrative rather than systematic approach was chosen deliberately. The field spans heterogeneous tasks, acquisition, segmentation, deformation imaging, valvular and vascular assessment, and outcome prediction, evaluated against disparate reference standards and reported with non-comparable metrics, such that a pooled quantitative synthesis would be neither feasible nor meaningful; this limitation is revisited in Section 6.7. To minimize selection bias, the underlying literature was identified through a structured search of PubMed/MEDLINE, Scopus, Web of Science, and Google Scholar (January 2016–May 2026), combining Medical Subject Headings and free-text terms. Search strings paired AI-related terms (“artificial intelligence”, “machine learning”, “deep learning”, “convolutional neural network”, “foundation model”, and “transformer”) with ultrasound- and domain-specific terms (“echocardiography”, “cardiovascular ultrasound”, “Doppler”, “speckle-tracking”, “global longitudinal strain”, “left ventricular ejection fraction”, “valvular heart disease”, “aortic stenosis”, “carotid plaque”, “stroke risk”, “point-of-care ultrasound”, and “risk prediction”) using the Boolean operators AND and OR. Peer-reviewed original studies, meta-analyses, reviews, and consensus or guideline documents published in English were prioritized, complemented by seminal earlier contributions and by screening of reference lists; non-peer-reviewed material and studies lacking either an AI or a cardiovascular-ultrasound component were not considered. Explicit inclusion criteria were peer-reviewed original studies, meta-analyses, systematic or narrative reviews, and consensus or guideline documents, published in English, that applied an AI or machine-learning method to cardiovascular ultrasound; explicit exclusion criteria were non-peer-reviewed material (preprints, conference abstracts, and editorials), non-English publications, and studies lacking either an AI or a cardiovascular-ultrasound component.

## 2. Advanced Cardiovascular Ultrasound: Fundamentals

### 2.1. Principles of Cardiac and Vascular Doppler Ultrasound

Doppler ultrasound remains a principal noninvasive method for structural and functional cardiovascular evaluation. Exploiting the Doppler effect, the frequency shift of reflected ultrasound proportional to the velocity and direction of moving red blood cells, its pulsed-wave, continuous-wave, and color-flow modalities enable real-time assessment of blood-flow direction, velocity, and turbulence and the derivation of intracardiac pressures, transvalvular gradients, and vascular resistance, extending ultrasound beyond purely anatomic assessment [3].

### 2.2. Modern Ultrasound Techniques

Tissue Doppler imaging (TDI) provides direct measurement of myocardial velocities and is central to diastolic-function assessment. STE tracks acoustic markers within the myocardium in an angle-independent manner, quantifying deformation through global longitudinal strain (GLS) and regional strain, indices particularly valuable for detecting subclinical dysfunction [2,3]. Three-dimensional (3D) echocardiography provides volumetric quantification of cardiac chambers without geometric assumptions; standardized recommendations underpin reproducible left ventricular (LV) and left atrial (LA) volume and LV ejection fraction (LVEF) measurement [16], and automated 3D analysis shows strong agreement with manual 3D tracings and cardiac magnetic resonance (CMR) while reducing acquisition and analysis time [17]. Contrast-enhanced ultrasound improves endocardial delineation and perfusion assessment, and vascular ultrasound characterizes intima-media thickness, atherosclerotic plaque, and arterial stiffness. Advanced deformation methods extend this further: three-dimensional and layer-specific strain and non-invasive myocardial work [18,19], which incorporates afterload via blood-pressure-derived pressure–strain loops, provide load-aware descriptors of contractility, but their computational complexity and vendor dependence have limited routine use, making them natural targets for automation.

### 2.3. Limitations of the Conventional Approach

Despite these advances, conventional cardiovascular ultrasound remains constrained by several factors. Acquisition and interpretation are highly operator-dependent, producing substantial inter- and intra-observer variability [4]. A specific and clinically important source of error is the acquisition of standard imaging planes: apical foreshortening and off-axis or non-standard views distort chamber geometry and systematically bias volume, LVEF, and strain measurements. Two-dimensional volume and LVEF estimation additionally relies on geometric assumptions and is load-dependent, and manual tracing is time-consuming and variable, considerations that motivated standardized reference values [16]. Conventional parameters also offer limited sensitivity for subclinical impairment, and the growing volume and complexity of imaging data increasingly exceed subjective visual assessment [5]. Collectively, these limitations have motivated automated, data-driven tools that standardize measurement and extract clinically meaningful patterns beyond conventional interpretation [20] (Table 1).

## 3. Artificial Intelligence in Cardiovascular Imaging

### 3.1. Fundamental Concepts, Architectures, and Evaluation

AI refers to computational systems that perform tasks normally requiring human intelligence. Within AI, ML denotes algorithms that learn patterns directly from data; DL, a subset based on multilayered neural networks, learns hierarchical image features and underlies most modern image-analysis applications [6,7]. The convolutional neural network (CNN) has been the predominant architecture for echocardiographic image and video analysis [8], with learning typically supervised (expert-labeled data) or unsupervised (discovering structure without labels).

The architectural landscape has, however, diversified considerably. Vision transformers (ViTs) and hybrid CNN–transformer models, such as TransUNet [21] and SegFormer [22], capture long-range spatial dependencies and now match or exceed pure CNNs on echocardiographic segmentation, while promptable segmentation models derived from the Segment Anything Model (SAM) [23] and its ultrasound adaptations have broadened generalization. In parallel, self-supervised and masked-autoencoding strategies reduce dependence on scarce expert labels and provide the pretraining backbone for the foundation models discussed in Section 3.4. Accordingly, statements about “CNN-based” systems should be read as historically dominant rather than architecturally exhaustive. Reported benchmark performance, however, does not necessarily transfer to routine practice: segmentation and quantification models are sensitive to multi-vendor domain shift, to acoustic and hardware differences across ultrasound platforms, and to image artifacts such as reverberation, shadowing, and signal dropout, all of which can degrade robustness at deployment and motivate multi-vendor training and external validation [24] (Section 6.2). In practice, this argues for reporting external, multi-vendor test performance alongside internal results, and for pre-deployment testing on the specific hardware and patient populations a tool will encounter, rather than assuming that benchmark accuracy generalizes.

**Figure 1:**
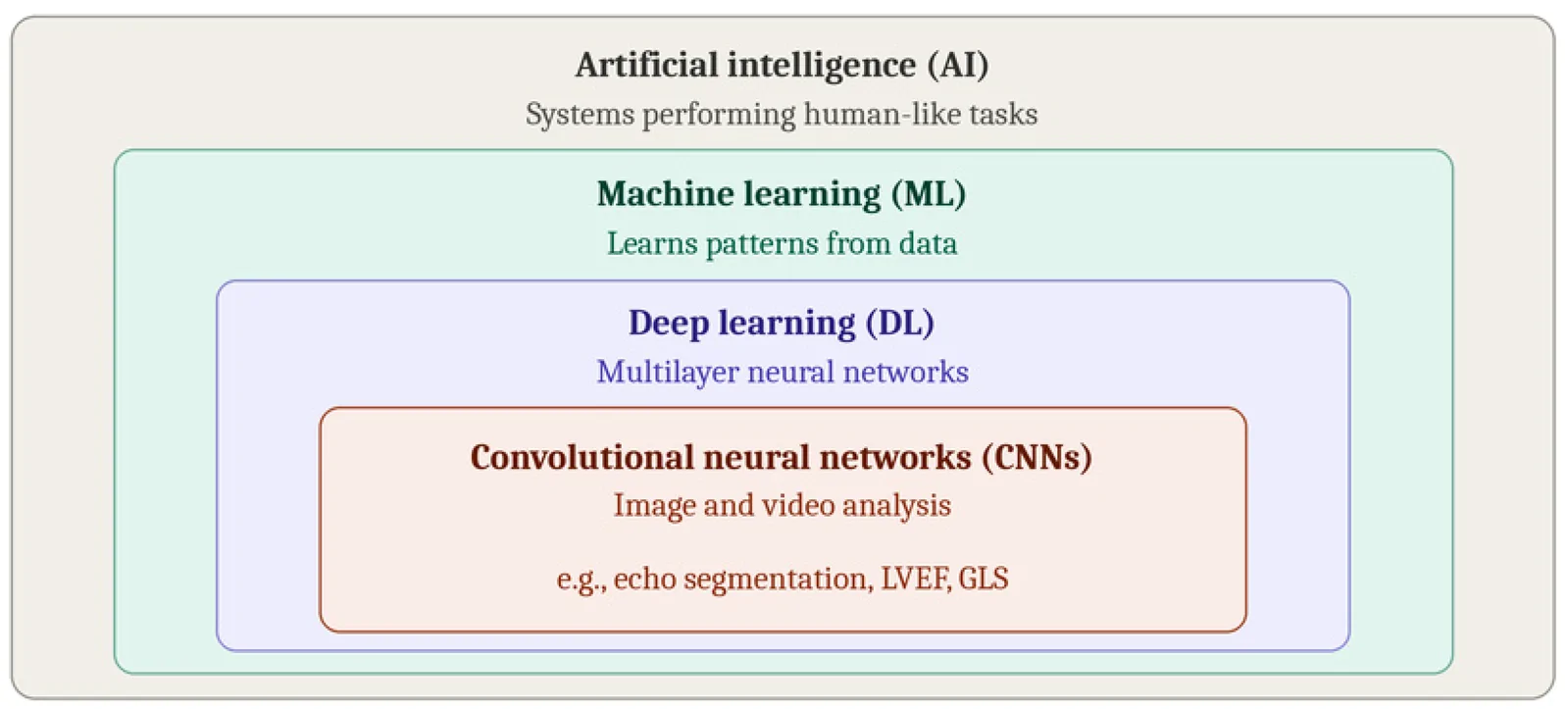
Hierarchical relationship between AI, machine learning, deep learning, and convolutional neural networks.

Model performance is reported with familiar diagnostic metrics, sensitivity, specificity, and the area under the receiver operating characteristic curve (AUC), and, for continuous measurements, correlation and mean absolute error (MAE) against expert or reference-standard values. For segmentation tasks specifically, spatial-overlap metrics, above all the Dice similarity coefficient (DSC) and, secondarily, the Hausdorff distance, are the appropriate standard and are reported alongside downstream functional agreement wherever available in this review. A clear separation between training, validation, and external test datasets is essential for generalizability [25,26]. The most informative evidence comes from external validation on data from institutions, scanners, and populations not seen during development and from temporal validation on prospectively acquired studies; agreement confined to an internal test split drawn from the same source is a weak indicator of real-world performance. The present review treats AI models as clinical tools and focuses on their tasks, quantitative performance, and limitations rather than their internal architecture (Figure 1).

### 3.2. The AI Pipeline in Cardiovascular Ultrasound

**Figure 2:**
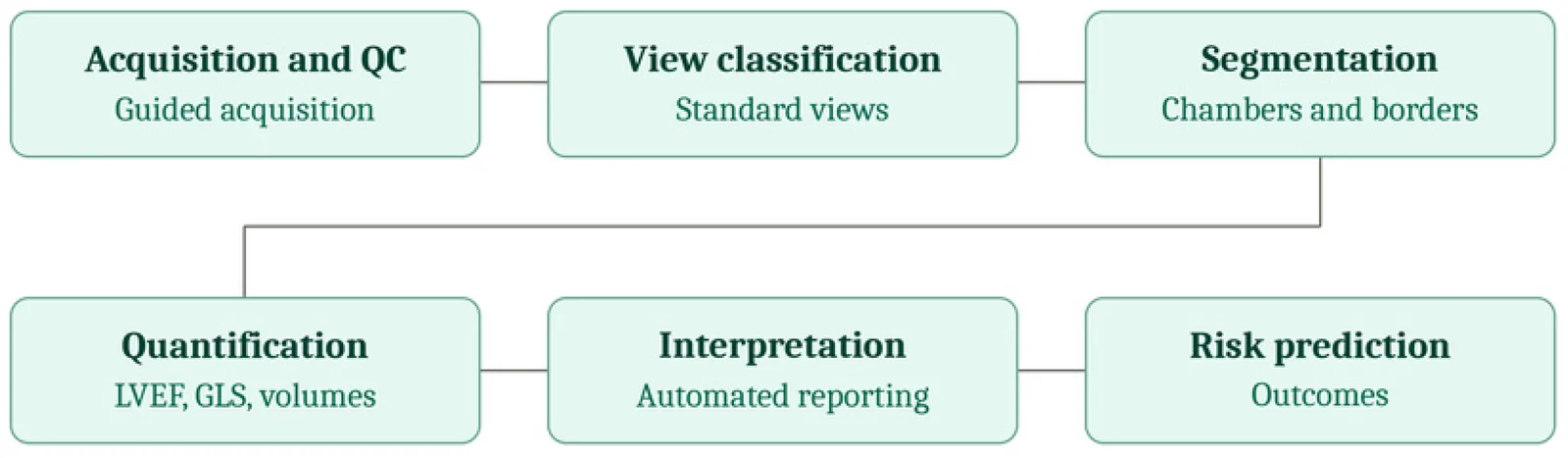
The AI pipeline in cardiovascular ultrasound, from image acquisition to risk prediction.

Rather than acting at a single step, AI can be embedded across the entire echocardiographic workflow (Figure 2). At acquisition, DL algorithms provide real-time scanning guidance and automated quality control (QC), enabling even novices to obtain diagnostic-quality images and, importantly, mitigating foreshortening and off-axis acquisition through view-specific probe prompts and per-frame quality scoring [14]. Acquired studies are automatically sorted by view, with classifiers identifying standard views at expert-level accuracy [27]; segmentation then delineates chambers and endocardial borders frame by frame [28], from which parameters such as LVEF are quantified [29]. Quality control operates throughout: models can score image adequacy, reject foreshortened or off-axis frames, and select the optimal cardiac cycles for measurement, ensuring that downstream quantification is performed on diagnostically sufficient data rather than on whatever frame is presented. Beyond measurement, integrated systems interpret studies end to end [30], and multitask frameworks combine disease detection, lesion localization, and phenogrouping within a single model [31,32]. Finally, extracted features can feed predictive models estimating cardiovascular risk and outcomes [13]. This stepwise integration reframes ultrasound from a descriptive examination into an integrated, decision-supporting platform.

### 3.3. Advantages of AI in Cardiovascular Ultrasound

AI confers several clinically relevant advantages. By automating measurement it markedly reduces inter- and intra-observer variability and improves reproducibility [4,10]; it accelerates and standardizes the workflow, lowering analysis time and supporting higher throughput [8]; through acquisition guidance it democratizes access to diagnostic-quality imaging, extending POCUS to nonexpert users and resource-limited settings [14]; and it can enhance sensitivity for subclinical disease by detecting subtle patterns and imaging biomarkers [9]. Representative tasks, benefits, and supporting evidence are summarized in Table 2.

### 3.4. Emerging Architectures: Foundation Models, Vision–Language Models, and LLMs

A decisive recent shift is the move from narrow, single-task, single-view networks toward large-scale foundation models that learn general-purpose representations from millions of studies and transfer across many downstream tasks. EchoCLIP, a vision–language foundation model trained on more than one million echocardiographic videos paired with expert reports, performed well across diverse interpretation benchmarks without task-specific training, estimating LVEF with an MAE of 7.1% on external validation and identifying implanted intracardiac devices with high accuracy [34]. EchoPrime extended this paradigm to a multi-view, view-informed, video-based model trained on over twelve million video–report pairs; it natively synthesizes information across the standard views of a complete study, matches or exceeds task-specific models across cardiac structures, and has been released with open code and weights [35]. Complementary multitask foundation approaches, exemplified by PanEcho, which couples a convolutional encoder with a temporal transformer to produce dozens of clinical outputs from any view, demonstrate complete, view-agnostic interpretation within a single model [32].

Beyond image analysis, large language models (LLMs) are beginning to support structured report generation, extraction of findings from free-text echocardiographic reports, guideline-concordant severity grading, and conversational clinical decision support, and vision–language architectures make image-to-text search and preliminary automated interpretation feasible [34]. Collectively, these models point toward general-purpose echocardiographic interpretation rather than a proliferation of narrow tools; their principal current limitations, dependence on very large paired datasets, uneven external validation, and interpretability, are discussed in Section 6.

The appeal of this paradigm is that a single pre-trained model can be adapted to many downstream tasks with comparatively little labeled data, mitigating the annotation bottleneck that constrains supervised learning and improving robustness through exposure to vast, heterogeneous data. The same scale, however, concentrates risk: biases, spurious correlations, or gaps in the pre-training corpus propagate to every downstream application, and the computational and data requirements raise questions of access, cost, and environmental impact. Rigorous, task-specific external validation therefore remains essential even for models that perform impressively in aggregate, and the transparency of training data becomes a governance concern in its own right.

### 3.5. Public Datasets and Benchmarks

Progress in echocardiographic AI has been shaped decisively by the availability of large, curated, publicly accessible datasets, which enable reproducible benchmarking and external validation. The CAMUS dataset provides 2D apical two- and four-chamber sequences with expert endocardial and epicardial tracings and remains the reference benchmark for LV segmentation [28]. EchoNet-Dynamic contributes more than ten thousand apical four-chamber videos with LVEF labels and frame-level tracings and underpinned the first video-based beat-to-beat EF model [33]; the companion EchoNet-LVH dataset adds parasternal long-axis videos annotated for wall thickness and chamber dimensions, supporting research on hypertrophy and its etiologies [36]. Complementary resources extend these benchmarks to additional views, pediatric populations, valvular disease, and multimodal linkage with population cohorts such as the UK Biobank. Table 3 summarizes representative datasets. Two caveats recur: many benchmarks derive from a small number of academic centers using particular scanners, which constrains external generalizability (Section 6.2), and label quality is bounded by the very inter-observer variability AI seeks to reduce.

### 3.6. Generative Models and Synthetic Data

Generative adversarial networks (GANs) and, more recently, diffusion models can synthesize realistic echocardiographic images and videos with controllable anatomy and function, offering a route to augment scarce or imbalanced datasets, to generate privacy-preserving shareable data, and to simulate rare pathologies for training [40]. Conditioned video-diffusion models can, for example, produce sequences with a prescribed ejection fraction, supporting data augmentation for function estimation. These opportunities carry a specific risk already discussed in Section 6.2: when models are trained recursively on their own synthetic outputs, fidelity and diversity can progressively degrade, the phenomenon of model collapse [41]. Responsible use therefore couples synthetic augmentation with a foundation of curated real-world data and explicit provenance tracking.

### 3.7. Explainability and Interpretability

Because many high-performing models are opaque, interpretability techniques are increasingly regarded as prerequisites for clinical trust and regulatory acceptance [7,10]. Post hoc saliency methods, class-activation mapping and gradient-based attribution, highlight the image regions most influential to a prediction, allowing clinicians to verify that a model attends to anatomically plausible structures rather than spurious artifacts such as text overlays or acquisition markers. Attention weights in transformer-based and vision–language models offer an analogous, architecture-native window onto model reasoning, and prototype- or concept-based approaches attempt to express decisions in clinically meaningful terms. Interpretability is not merely reassuring: it supports error analysis, exposes dataset shortcuts and bias, and is central to the human oversight that emerging regulation requires. Nonetheless, saliency maps can be unstable and are not a substitute for rigorous external validation; interpretability and empirical performance must be evaluated together. The stakes are highest in time-critical presentations such as acute myocardial ischemia, cardiac tamponade, or cardiogenic shock, where clinicians cannot act on an uninterpretable output without rapid visual verification; architecture-native interpretability, including faithful feature attribution and validated saliency or attention overlays, is therefore needed to support immediate clinical trust and safe use at the bedside. Recent syntheses distinguish post hoc explanation, applied to an already-trained black box, from intrinsically interpretable, ante hoc designs and catalogue the method families now used in cardiovascular imaging, including gradient- and perturbation-based saliency, attention visualization, and concept-, prototype-, and counterfactual-based reasoning, each offering different guarantees of faithfulness and clinical plausibility [42]. A caution echoed across specialties is that explanations must themselves be validated: a plausible-looking heatmap is not evidence of correct reasoning, and unstable or misleading explanations can foster misplaced trust, so quantitative assessment of explanation fidelity and robustness should accompany deployment in high-stakes cardiac and respiratory care [43]. Interpretability is thus best treated not as an afterthought but as a design goal, reported to a defined standard and evaluated alongside diagnostic performance. In the cardiovascular setting specifically, interpretability that is faithful and clinically meaningful is increasingly regarded as a precondition for regulatory acceptance and for the clinician trust on which real-world adoption ultimately depends [42].

## 4. Clinical Applications of AI in Cardiovascular Ultrasound

**Figure 3:**
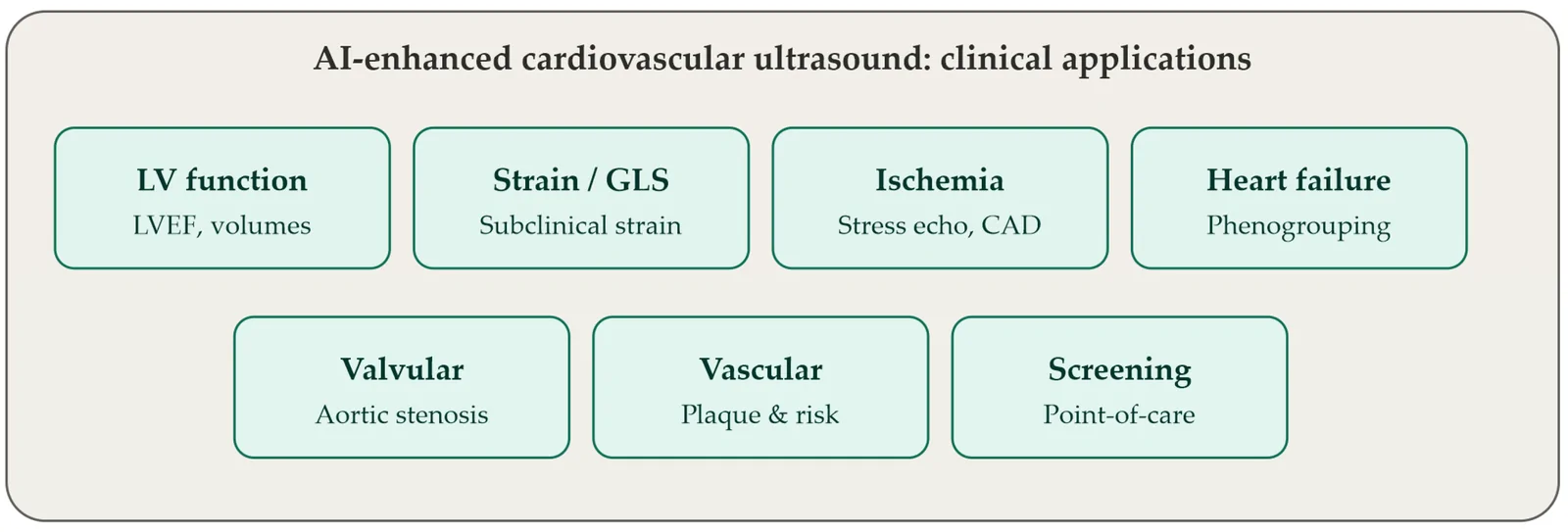
Principal clinical applications of AI-enhanced cardiovascular ultrasound.

Building on the workflow above, AI has been applied across the spectrum of cardiovascular ultrasound. This section reviews the principal clinical domains, beginning with LV function and myocardial deformation, where the evidence base is most mature, and proceeding through ischemic, valvular, and vascular disease; the cardiomyopathies; diastolic function; fetal and congenital heart disease; and right-heart and atrial assessment (Figure 3). These domains differ markedly in maturity, from tasks approaching routine clinical use to those still at proof-of-concept, a heterogeneity made explicit in the synthesis of Section 6.

### 4.1. Left Ventricular Function and Automated Quantification

Automated quantification of LV systolic function is among the most mature applications of AI in echocardiography. CNN-based segmentation of the LV across the cardiac cycle enables calculation of end-diastolic volume (EDV), end-systolic volume (ESV), and LVEF without manual tracing [28]. Quantitative performance is now well characterized: on the publicly available CAMUS dataset, encoder–decoder networks reproduced expert LV volumes with a mean correlation of 0.95 and an MAE of 9.5 mL, LVEF with a correlation of 0.80 and an MAE of 5.6% and achieved LV-endocardial Dice similarity coefficients of approximately 0.94 (end-diastole) and 0.92 (end-systole), approaching inter-observer agreement [28]. The video-based EchoNet-Dynamic model reported an LV Dice of 0.92, an LVEF MAE of 4.1%, and classification of heart failure with reduced ejection fraction with an AUC of 0.97 [33]. Dedicated algorithms can estimate ejection fraction directly, mimicking expert readers without explicit volume measurement [29], and fully automated pipelines produce measurements within the range of inter-observer variability in routine practice [30]. Beyond agreement with human readers, the value of reproducible automated function lies in its prognostic reach: video-based models capture beat-to-beat variation that single manual measurements miss, and automatically derived structural and functional parameters have been linked to downstream cardiovascular outcomes, positioning automated quantification as a substrate for risk prediction rather than an end in itself [33,44].

Three-dimensional approaches further refine quantification: automated 3D analysis of LV and LA volumes and LVEF correlated strongly with manual 3D tracings (r = 0.87–0.96) and CMR (r = 0.84–0.95), with low test–retest variability [17]. Comparable accuracy with substantial time savings has been reported for combined automated LVEF and strain [45], and DL systems derive functional parameters across heterogeneous, real-world studies [44]. Representative LV-function algorithms and their quantitative performance, including spatial-overlap metrics, are summarized in Table 4.

### 4.2. Myocardial Deformation: Strain and Global Longitudinal Strain

GLS derived from STE is more sensitive than LVEF for early myocardial dysfunction but has historically been limited by vendor- and operator-dependent variability [2]. AI addresses this directly: DL methods measure LV strain automatically with strong agreement to reference STE while substantially reducing analysis time [46,47]. Multiple test–retest studies show that AI improves the precision and reproducibility of strain relative to conventional analysis [48,49], with repeatability and accuracy comparable to experienced echocardiographers [50]. AI has been leveraged to standardize GLS across vendors and readers [51], and models trained on large open collaborative datasets produce GLS estimates in close agreement with human experts [52]. Crucially, an automated strain algorithm has been externally validated on independent, multi-vendor cohorts, an essential step toward generalizable deployment that most strain studies have not undertaken [53]. Clinically, AI-derived strain has been applied to surveillance of cancer therapy-related cardiac dysfunction (CTRCD), where reproducible detection of subclinical decline is essential for timely intervention [54]. Representative strain studies are summarized in Table 5.

### 4.3. Ischemic Heart Disease

Echocardiographic ischemia detection relies on identifying regional wall-motion abnormalities (RWMAs), a task with well-documented inter-observer variability. DL models detect RWMAs from standard views with accuracy approaching experienced readers [55]. The greatest gains have come in stress echocardiography for coronary artery disease (CAD): Upton et al. developed an automated image-processing and ML pipeline that extracted geometric and kinematic features from stress echocardiograms and identified patients with severe CAD on invasive angiography, with performance confirmed in an independent cohort [56]; complementary work shows that AI-assisted LV assessment increases the diagnostic accuracy of stress echocardiography relative to conventional interpretation [57]. By standardizing recognition of subtle, transient functional changes, AI may reduce the operator dependence that has limited reproducibility of ischemia assessment. Reader studies indicate that AI augmentation most benefits lower-performing and less-experienced operators, raising their accuracy toward that of experts [56]. Importantly, this domain has also produced prospective randomized evidence: as discussed in Section 6.4, the first multicenter randomized trial of AI-augmented stress-echocardiography interpretation returned a nuanced result, tempering expectations that reader-study gains translate automatically into improved care pathways [58].

### 4.4. Heart Failure

AI has shown particular promise in heart failure (HF), where phenotypic heterogeneity complicates diagnosis. ML applied to standard 2D parameters distinguishes physiological from pathological hypertrophy, for example, separating athlete’s heart from hypertrophic cardiomyopathy (HCM) [59], and cognitive ML has differentiated constrictive pericarditis from restrictive cardiomyopathy [60]. In HF with preserved ejection fraction (HFpEF), unsupervised learning has derived prognostically distinct phenogroups from dense clinical and echocardiographic data [61]. This capability has translated to the clinic: the EchoGo Heart Failure platform, which detects HFpEF from a single echocardiographic view, obtained FDA clearance as a breakthrough device (Section 5.5). Phenogrouping has also been linked to therapeutic response, identifying patients more likely to benefit from cardiac resynchronization therapy (CRT) [62], and multitask DL supports disease-specific recognition and phenogrouping within a single model [31]. Finally, integrating echocardiographic measurements with electronic health record (EHR) data has predicted survival more accurately than conventional scores [63], underscoring the prognostic value of AI-augmented echocardiography in HF [10]. The broader significance of phenogrouping lies in reframing heterogeneous syndromes such as HFpEF as collections of mechanistically distinct subtypes: unsupervised clustering of dense phenotypic data can reveal subgroups with different natural histories and, critically, different responses to therapy, transforming a one-size-fits-all diagnosis into a substrate for individualized treatment [61]. Realizing this clinically will require that data-derived phenogroups prove stable and reproducible across cohorts and that their therapeutic implications be confirmed prospectively.

### 4.5. Valvular Heart Disease

Valvular heart disease (VHD) assessment, traditionally dependent on Doppler-derived hemodynamics, is increasingly amenable to AI [64]. A notable example is aortic stenosis (AS): Holste et al. developed a DL model detecting severe AS directly from limited 2D views without spectral Doppler, with high discrimination and the ability to retrospectively flag potentially missed disease [65]. Because such models operate on grayscale views, they suit screening and point-of-care contexts where full Doppler quantification is impractical; commercially, automated guideline-based AS grading (Vmax, mean gradient, aortic-valve area, and severity) is now available in CE-marked/FDA-cleared platforms (Section 5.5). More broadly, AI for VHD aims to standardize severity grading and reduce the expertise required for accurate assessment [64]. Beyond aortic stenosis, automated analysis is being extended to mitral and tricuspid regurgitation, where severity grading is notoriously subjective and integrates multiple qualitative and quantitative signs; consistent, reproducible quantification could reduce the inter-observer variability that complicates the timing of intervention. As with other domains, the decisive step will be prospective demonstration that AI-standardized grading improves clinical decisions rather than merely agreeing with expert reads.

### 4.6. Carotid and Peripheral Vascular Disease

Carotid ultrasound provides a noninvasive window onto subclinical atherosclerosis, and ML has been applied extensively to characterize plaque and stratify stroke risk. Early ML classified echolucent plaque morphology to stratify stroke risk [66], and tissue-characterization frameworks established feasibility of automated analysis [67]. DL has since improved characterization and segmentation: CNN-based models characterized internal carotid plaque [68], and 3D-optimized systems advanced automated risk stratification [69]. For quantitative burden, DL segmentation of B-mode images enabled automated total plaque area (TPA) measurement with strong agreement to manual delineation (r ≈ 0.99) and segmentation times of milliseconds per plaque [70]. Additional models addressed plaque detection and classification [71] and integrated carotid imaging into broader risk tools: combining conventional risk factors, carotid ultrasound, and intraplaque neovascularization, deep-learning frameworks have predicted coronary artery disease and enabled low-cost, office-based risk stratification [72,73]. Population data reinforce prognostic value: in the Multi-Ethnic Study of Atherosclerosis, carotid measures were among the strongest ML-identified predictors of stroke [13], illustrating how automated vascular phenotyping can feed directly into population-level risk models. Because carotid ultrasound is inexpensive, radiation-free, and widely available, reproducible automated plaque quantification is particularly attractive for scalable, opportunistic screening of subclinical atherosclerosis. A comprehensive overview, including datasets and methodological challenges, is available elsewhere [74]. Representative carotid studies are summarized in Table 6.

### 4.7. Cardiovascular Screening and Point-of-Care Ultrasound

A recurring theme is AI’s potential to extend cardiovascular ultrasound beyond the expert laboratory. Real-time acquisition guidance enables novices to obtain diagnostic-quality echocardiograms [14], and AI-assisted focused cardiac ultrasound has shown useful diagnostic accuracy in primary care [15]. Algorithms operating on limited grayscale views, such as those for AS detection [65], and multitask systems validated on handheld devices [31] make opportunistic screening feasible at the point of care. Coupled with telemedicine and remote interpretation, these capabilities could broaden access in resource-limited settings, supporting earlier detection and a shift toward preventive, population-level care. The same combination, AI-guided acquisition on handheld devices plus automated interpretation, also enables opportunistic case-finding for under-recognized but treatable conditions such as cardiac amyloidosis and HFpEF during unrelated encounters, effectively turning a brief bedside scan into a screening opportunity [75]. Realizing this at scale, however, requires validation in the lower-quality images and unselected populations characteristic of point-of-care settings, where performance may differ from that reported in curated laboratory cohorts. In addition, AI is extending point-of-care assessment beyond the heart to thoracic ultrasound: automated quantification of B-lines and pulmonary-congestion scoring can complement echocardiography in acute heart-failure triage and cardiopulmonary evaluation, and deep-learning algorithms already detect and count B-lines in agreement with expert readers [76,77,78].

### 4.8. Cardiomyopathies: Hypertrophic Cardiomyopathy and Cardiac Amyloidosis

Increased LV wall thickness is common, but distinguishing its causes, hypertension, hypertrophic cardiomyopathy (HCM), and cardiac amyloidosis, is clinically consequential and frequently delayed. AI is well suited to this problem because the discriminating features are subtle and distributed across the cardiac cycle. Trained on the EchoNet-LVH dataset, a video-based deep-learning workflow measured intraventricular septal thickness, LV internal diameter, and posterior-wall thickness with mean absolute errors of roughly 1.2–2.4 mm and classified cardiac amyloidosis and HCM as distinct from other causes of hypertrophy with areas under the curve of 0.83 and 0.98, respectively, retaining discrimination (amyloidosis AUC 0.79; HCM AUC 0.89) on external validation [36]. Because amyloidosis and HCM are under-recognized yet increasingly treatable, such algorithms are attractive as opportunistic screens applied retrospectively to imaging archives. Extending this to the point of care, a multicenter study demonstrated AI-guided detection of under-recognized cardiomyopathies on handheld cardiac ultrasonography, supporting community-level case-finding [75]. In HCM specifically, machine-learning wall-thickness measurement has matched or exceeded human test–retest performance, improving the consistency of a measurement on which diagnosis and risk stratification directly depend [79]. Because diagnosis triggers disease-specific therapy, and, for transthyretin amyloidosis and obstructive HCM, increasingly effective treatments, reproducible AI phenotyping has immediate management implications.

### 4.9. Diastolic Function and Automated Doppler Interpretation

Assessment of diastolic function is algorithmically complex, integrating multiple 2D and Doppler measurements, and is a common source of inter-observer disagreement. Deep-learning workflows now classify, segment, and annotate both 2D videos and spectral/tissue Doppler modalities: a multicohort study developed a fully automated pipeline for systolic and diastolic function that reproduced expert interpretation across international cohorts [80], and dedicated models grade diastolic dysfunction directly from echocardiographic data with agreement approaching that of expert readers [81]. By standardizing Doppler measurement and the rule-based integration that follows, these tools address a domain where guideline algorithms are intricate and reproducibility has been limited, an important enabler for scalable HFpEF screening (Section 4.4).

### 4.10. Fetal and Congenital Heart Disease

Congenital heart disease is the most common birth defect, yet prenatal detection is highly variable and, in routine practice, sensitivity for complex lesions can be as low as 30% [82]. Deep learning offers a route to standardize fetal cardiac screening. In a landmark study, an ensemble of neural networks trained on more than 100,000 images identified recommended cardiac views, distinguished normal from abnormal hearts, and calculated standard fetal cardiothoracic measurements, achieving an AUC of 0.99 with 95% sensitivity and 96% specificity for complex congenital heart disease on a large internal test set [82]. Comparable approaches are being extended to first-trimester screening and to pediatric echocardiography, domains constrained by anatomical complexity, lesion rarity, and a global shortage of specialized expertise, precisely the conditions under which reproducible AI support is most valuable.

### 4.11. Right Ventricular and Atrial Assessment

Although the left ventricle has received the most attention, AI is increasingly applied to structures whose manual assessment is especially challenging. Automated analysis of right-ventricular size and function, historically limited by complex geometry and load dependence [83,84], and of left- and right-atrial volumes and strain [85] extends reproducible quantification to chambers where conventional measurement is time-consuming and variable. These capabilities broaden the multiparametric substrate available to the multimodal predictive models discussed in Section 5 and are being incorporated into the comprehensive, multi-view foundation models that report dozens of structural and functional parameters from a complete study [32,35].

### 4.12. Contrast Echocardiography and Myocardial Perfusion

Contrast echocardiography improves endocardial border delineation in technically difficult studies and, with myocardial contrast techniques, allows assessment of perfusion. Both applications are natural targets for AI: automated opacification scoring can guide contrast administration and image optimization, while quantitative perfusion analysis, tracking contrast wash-in and wash-out, is measurement-intensive and observer-dependent, and therefore stands to benefit from automated, reproducible quantification. By improving border detection in poor-quality images, AI-assisted contrast interpretation may also extend reliable functional assessment to the very patients in whom conventional analysis is least dependable, complementing the automated quantification pipelines described above [9]. Evidence here is less mature than for chamber quantification or strain, and dedicated validation of AI in contrast and perfusion imaging remains an open and clinically important direction.

## 5. Imaging Biomarkers and Predictive, Personalized Medicine

### 5.1. The Concept of Precision Cardiology

Precision cardiology seeks to tailor prevention, diagnosis, and treatment to the individual rather than to population averages [11]. Realizing this requires tools that convert routine imaging into quantitative, reproducible, and prognostically meaningful information, precisely the capability AI brings to cardiovascular ultrasound [12]. By transforming descriptive images into structured data, AI positions echocardiography and vascular ultrasound as sources of digital biomarkers integrable with clinical, laboratory, and genetic data for individualized decision-making [6,9]. The shift is conceptual as much as technical: whereas conventional reporting yields qualitative impressions and a handful of measurements, AI can extract dense, quantitative, reproducible representations of cardiac structure and function from every study. Aggregated across populations, these representations support data-driven definitions of normality and disease that are tailored to age, sex, and body habitus rather than applied uniformly, and at the individual level they enable a patient’s imaging phenotype to be interpreted against the most relevant reference group, a prerequisite for genuinely personalized cardiovascular care.

### 5.2. AI-Derived Imaging Biomarkers of Subclinical Disease

A central contribution of AI is the reliable extraction of imaging biomarkers sensitive to subclinical disease. AI-derived GLS detects early myocardial dysfunction before LVEF declines, with the reproducibility needed for serial monitoring [46]. In the vascular system, automated quantification of plaque burden such as TPA captures subclinical atherosclerosis and its response to therapy [70]. These biomarkers extend the diagnostic reach of ultrasound toward preclinical stages, and population studies confirm that such measures carry independent prognostic weight [13]. Their clinical value depends on reproducibility across time and observers, precisely where AI contributes most, since serial monitoring requires that a measured change reflect true biological change rather than measurement noise. Embedding them into routine workflows is increasingly feasible as AI reduces the expertise and time previously required [10].

### 5.3. Multimodal Predictive Models and Risk Stratification

The prognostic value of imaging biomarkers is amplified when combined with other data streams. ML models trained on deeply phenotyped cohorts, integrating imaging, electrocardiographic, and biomarker data, have outperformed conventional risk scores in predicting cardiovascular events [13], and coupling echocardiographic measurements with EHR data has improved survival prediction [63]. Deep learning further enables analysis of raw images and high-dimensional inputs that traditional statistics cannot readily accommodate [86]. A key advantage of multimodal fusion is complementarity: echocardiographic structure and function, electrocardiographic electrical signatures, laboratory biomarkers, and longitudinal EHR trajectories capture partially independent facets of cardiovascular risk, so their combination can outperform any single modality. Foundation and vision–language models further enable joint reasoning over images and text, laying the groundwork for systems that ingest a complete study alongside the clinical record [34,35]. Realizing this potential nonetheless demands careful methodology, harmonized data, guarding against leakage between modalities, and calibration across sites before multimodal scores can be trusted for individual decisions. Collectively, these multimodal models move risk assessment from single-parameter thresholds toward individualized, data-driven prediction [11].

### 5.4. Therapy Personalization and Prognosis

**Figure 4:**
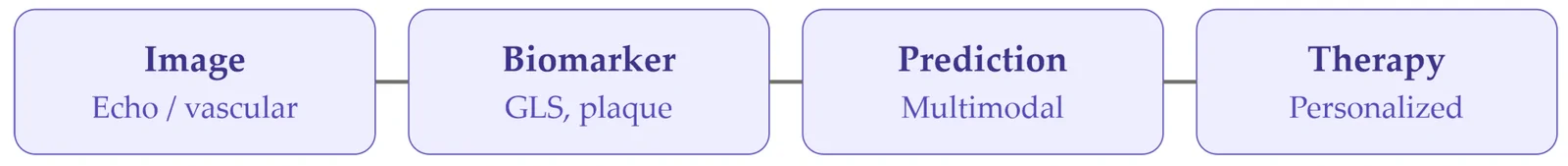
The precision-cardiology pathway: from image to biomarker, risk prediction, and personalized therapy.

Ultimately, AI-derived stratification can inform therapy. ML-defined phenogroups in HFpEF and broader HF have distinguished patients with different prognoses and different likelihoods of benefiting from interventions such as CRT [61,62]. In oncology, AI-based strain surveillance supports early detection of CTRCD, enabling timely cardioprotection [54]. These examples illustrate a coherent pathway, from image, to reproducible biomarker, to risk prediction, to personalized therapy, that defines the contribution of AI-enhanced ultrasound to precision cardiology (Figure 4) [10].

### 5.5. Clinically Approved AI Tools

The translation of these capabilities into practice is reflected in a growing number of regulatory-cleared products. AI-based acquisition guidance received early De Novo authorization from the U.S. Food and Drug Administration (FDA) and enables novice operators to obtain interpretable studies [14]; fully automated analysis platforms provide guideline-based LVEF, GLS, diastolic-function, and AS quantification with FDA clearance and CE marking; and dedicated tools address HFpEF and cardiac-amyloidosis detection, the latter now feasible from a single view. Representative approved tools are summarized in Table 7; regulatory status evolves rapidly and should be verified against current FDA and EU databases.

### 5.6. Longitudinal Monitoring and Serial Assessment

Much of the clinical value of precision cardiology lies not in a single measurement but in tracking change over time, where conventional imaging is especially vulnerable to measurement noise. Because a difference between serial studies is clinically actionable only if it exceeds measurement variability, the improved reproducibility of AI-derived indices is decisive for surveillance. This is most developed in cardio-oncology, where AI-derived strain enables reliable detection of subclinical decline during cancer therapy, supporting earlier cardioprotective intervention than LVEF-based monitoring allows [54]. Analogous applications include serial quantification of plaque burden to monitor atherosclerosis progression and response to lipid-lowering therapy [70], surveillance of ventricular function in chronic valvular and myocardial disease, and consistent GLS tracking across visits and vendors, feasible only when an algorithm has demonstrated stable, externally validated performance [53]. Reproducible automated measurement thus reframes echocardiography from a series of isolated snapshots into a coherent longitudinal signal.

## 6. Discussion

### 6.1. Synthesis: Maturity Versus Evidence

The applications reviewed span a wide spectrum of maturity. Some tasks, view classification, chamber segmentation, and LVEF quantification, are well validated and approaching routine use [28,29,30,33]. An intermediate tier, cardiomyopathy and amyloidosis detection, diastolic-function interpretation, aortic-stenosis screening, and fetal congenital-heart-disease screening, shows strong or externally validated performance but awaits prospective, outcome-linked confirmation [36,75,80,82]. Others, multimodal risk prediction, HFpEF phenogrouping, and therapy personalization, remain largely investigational [13,61]. Critically, prospective randomized evidence has begun to emerge with contrasting results: an early single-center blinded trial found initial AI assessment of ejection fraction non-inferior, and even superior, to sonographer assessment [88], whereas the first multicenter randomized trial, of AI-augmented stress echocardiography, returned a mixed result (Section 6.4) [58], a reminder that diagnostic-accuracy maturity and demonstrated clinical benefit are distinct milestones. A meta-analysis of DL in medical imaging confirmed high diagnostic accuracy across domains but underscored marked heterogeneity in study design and reporting [89]. Distinguishing what is ready for deployment from what remains a research endeavor is essential for realistic expectations; Table 8 summarizes the maturity and evidence level.

### 6.2. Technical Limitations: Generalizability, Reproducibility, and Data Integrity

Several technical limitations constrain adoption. Many DL models function as “black boxes”, offering limited insight into how outputs are reached, which hinders clinical trust and accountability [7,10]. Generalizability is a recurring concern that is often underappreciated: models achieving high accuracy on curated public datasets (e.g., CAMUS or EchoNet-Dynamic) frequently degrade when transferred to private, real-world clinical data acquired on different scanners, at different institutions, or with different acquisition protocols. This distribution shift, driven by vendor-specific image characteristics, probe settings, and population differences, means that a Dice coefficient or LVEF MAE reported on a benchmark cannot be assumed to hold at deployment, and multi-center, multi-vendor validation remains the exception rather than the rule. This vulnerability is compounded by dataset bias: training data that under-represent certain demographic groups may propagate or amplify performance disparities [25,26]. Evidence from other imaging domains is instructive: deep-learning models applied to chest radiographs have been shown to systematically under-diagnose disease in under-served populations [90], underscoring the need for demographically representative training data, pre-specified fairness auditing across subgroups, and transparent reporting of performance by sex, age, ethnicity, and body habitus before clinical deployment.

Reproducibility is a distinct and frequently neglected concern. Only a minority of clinically framed studies release their source code or trained network weights, and even fewer make their imaging data available, which limits independent verification and slows translation. Notable exceptions demonstrate the value of openness: the CAMUS dataset and the EchoNet-Dynamic model and data are publicly available [28,33], and recent foundation models such as EchoPrime have released code and weights [35]. Wider adoption of open-code, open-weight, and, where privacy permits, open-data practices, together with standardized reporting of spatial-overlap metrics (Dice and Hausdorff distance) alongside functional agreement, would substantially strengthen the field’s maturity and clinical readiness.

A forward-looking data-integrity concern is model collapse: as AI-generated images and synthetic data proliferate, models trained recursively on such outputs can progressively lose fidelity and diversity, colloquially termed “Habsburg AI”, with degradation compounding across generations [41]. Because synthetic augmentation is increasingly used to address data scarcity in echocardiography, curating high-quality real-world data and monitoring for collapse will be important safeguards. Finally, the heterogeneity of reference standards and performance metrics across studies complicates direct comparison and quantitative synthesis [89].

Methodological rigor in reporting is itself a recurring limitation. Many studies emphasize correlation or overall accuracy while omitting the metrics most informative for clinical use, Bland–Altman limits of agreement and intraclass correlation for continuous measurements, spatial-overlap indices for segmentation, and calibration and decision-curve analysis for classifiers. Agreement with a human reader, moreover, is not equivalent to correctness when the reference standard is itself variable, and impressive discrimination on a retrospective cohort does not establish that a model changes management or improves outcomes. Consistent, prespecified reporting of these quantities, alongside confidence intervals and subgroup breakdowns, is necessary before performance claims can be compared across studies or trusted for individual patients.

### 6.3. Clinical Validation, Regulation, and Ethics

Translating AI into practice requires rigorous clinical validation, ideally prospective, multicenter studies with external testing rather than retrospective, single-center development [10]. Regulatory frameworks are evolving to accommodate software that learns and updates: the FDA has articulated guiding principles for Good Machine Learning Practice (GMLP) [91] and lifecycle-management and marketing recommendations for AI-enabled device software functions, including predetermined change-control plans [92]; in Europe, such devices fall under the Medical Device Regulation (EU MDR 2017/745). The expanding roster of FDA-cleared and CE-marked tools (Table 7) demonstrates a viable regulatory pathway while also raising the bar for post-market surveillance. Regulatory authorization of AI-enabled medical devices has accelerated markedly, with more than a thousand AI/ML-enabled devices authorized by the FDA as of December 2024 and cardiovascular applications among the most represented categories after radiology [87]; this pace intensifies the need for robust post-market monitoring, real-world performance auditing, and mechanisms to detect degradation or drift after deployment. A recurring gap is that clearance typically rests on demonstrating diagnostic performance rather than improved patient outcomes, so regulatory approval should be read as a floor for safety and analytic validity rather than proof of clinical benefit. Ethical and legal considerations, data privacy, informed consent for data use, algorithmic accountability, and equitable access must be addressed in parallel to ensure responsible deployment [10]. Privacy-preserving paradigms such as federated learning, in which models are trained across institutions without centralizing patient data, offer a practical route to the large, diverse, multi-vendor datasets that generalizable models require while respecting data-governance constraints [93]. Equally important is active attention to demographic, socioeconomic, and population-level bias: representative datasets, pre-specified subgroup fairness auditing, and equity-focused development are needed so that AI narrows rather than widens disparities in cardiovascular care [94]. In parallel, the European Union’s Artificial Intelligence Act (Regulation (EU) 2024/1689) introduces a risk-based framework under which most diagnostic AI qualifies as high-risk and must satisfy requirements for data governance, transparency, human oversight, and post-market monitoring, complementing the EU MDR and the FDA frameworks above.

### 6.4. Prospective Validation and Real-World Evidence

Most evidence in this field remains retrospective and single-center, and the transition to prospective, outcome-linked validation is only beginning. The first randomized evidence came from a single-center, blinded, non-inferiority trial in which initial AI assessment of left ventricular ejection fraction was non-inferior, and by pre-specified testing superior, to sonographer assessment, requiring substantial cardiologist correction less often [88]. A subsequent landmark example is PROTEUS, the first prospective, multicenter randomized controlled trial of an AI device in cardiovascular imaging, which evaluated AI-augmented interpretation of stress echocardiography for referral to invasive coronary angiography across twenty UK centers [58]. Notably, AI-augmented decision-making did not meet the pre-specified non-inferiority endpoint overall, although the authors suggested potential benefit in lower-volume centers where operator experience is more limited. This nuanced result is instructive: strong retrospective and reader-study performance does not guarantee benefit when an algorithm is embedded in a real clinical pathway, where human–AI interaction, automation bias, and workflow factors shape outcomes. It reinforces that prospective trials with clinically meaningful endpoints, not only diagnostic-accuracy metrics, are essential, and that the design of human–AI collaboration is as important as model performance itself. Fully automated pipelines validated across international cohorts point in the same direction, demonstrating reproducibility beyond the developing institution while still requiring outcome-linked confirmation [80].

Robust evaluation is inseparable from standardized reporting. General frameworks for AI in health, such as the CONSORT-AI and SPIRIT-AI extensions for trials, DECIDE-AI for early clinical evaluation, CLAIM for imaging studies, and STARD-AI for diagnostic-accuracy studies [95] define the transparency expected of development and validation, while cardiovascular-imaging-specific criteria have been proposed to structure the evaluation of multimodal AI, including data provenance, external testing, fairness, calibration, and clinical utility [96]. Consistent adoption of such frameworks would directly address the heterogeneity in study design and reporting that has repeatedly limited comparability across studies [89] and would help distinguish genuine clinical readiness from favorable but non-generalizable benchmark performance.

### 6.5. Implementation, Workflow Integration, and Human–AI Interaction

Even a well-validated algorithm delivers no benefit unless it is integrated effectively into clinical workflows and used appropriately by clinicians. The PROTEUS trial, in which strong retrospective performance did not translate into a positive pathway outcome, illustrates that implementation, not accuracy alone, ultimately determines clinical impact [58]. This section considers the practical dimensions of translation.

#### 6.5.1. Workflow Integration and Quality Assurance

Realizing AI’s efficiency gains requires embedding models seamlessly into acquisition and reporting systems, ideally producing structured, editable outputs within existing platforms rather than in parallel applications. A distinctive challenge is lifecycle governance: unlike static devices, AI performance can drift as scanners, populations, and protocols evolve, so continuous post-deployment monitoring, periodic recalibration, and clear versioning are essential, considerations now reflected in regulatory expectations for predetermined change-control plans [92]. A prudent adoption pattern is “silent-mode” or shadow deployment, in which an algorithm runs alongside routine reporting so that its real-world performance and failure modes can be characterized before it influences care. Comprehensive, multitask systems that generate many measurements from a complete study simplify integration by consolidating what would otherwise be numerous single-purpose tools [32]. Practical integration also depends on interoperability: outputs must flow through established standards, DICOM Structured Reporting for measurements and HL7/FHIR for exchange with the electronic record so that AI results populate reports and registries without manual re-entry. Fragmented, proprietary outputs that sit outside the reporting system add clicks rather than removing them and are a common reason capable algorithms fail to gain clinical traction. Meeting recognized interoperability standards, DICOM Structured Reporting for measurements and HL7/FHIR for exchange with the electronic record and registries is therefore a practical prerequisite for deployment at scale, and pilots should confirm standards-conformant output before go-live.

#### 6.5.2. Human–AI Interaction and Automation Bias

How clinicians interact with AI output is as consequential as the output itself. Over-reliance (automation bias) can propagate algorithmic errors, whereas unwarranted distrust forfeits benefit; both depend on how, when, and with what uncertainty information is surfaced. Evidence suggests the largest gains accrue to less-experienced operators and lower-volume settings, where AI narrows the gap to expert performance, while adding little, or occasionally introducing noise, for experts [58]. Designing decision support that calibrates clinician trust, communicates confidence, and preserves clinician agency is therefore a research priority in its own right, and human-factor evaluation should accompany diagnostic-accuracy assessment.

#### 6.5.3. Education, Training, and Evolving Roles

Widespread adoption will reshape the roles of sonographers and cardiologists. Curricula must equip practitioners to understand model capabilities and limitations, recognize failure modes, and supervise automated measurements rather than accept them uncritically. A recognized risk is deskilling, erosion of manual competencies as automation expands, which argues for training strategies that maintain core skills alongside AI proficiency and for clear delineation of accountability when AI contributes to a diagnosis [97].

#### 6.5.4. Health Economics, Reimbursement, and Equitable Access

Sustainable deployment depends on demonstrating value and establishing reimbursement. Prospective evaluations increasingly incorporate health-economic endpoints since efficiency gains, avoided downstream testing, and earlier detection must be weighed against acquisition, integration, and monitoring costs. Access equity cuts both ways: by democratizing expertise, AI-guided acquisition and interpretation could extend quality cardiovascular imaging to under-served and resource-limited settings [14], yet without deliberate attention to representative data and affordable implementation, the same technologies could widen existing disparities. Aligning incentives, evidence, and equity is thus central to responsible scaling [10].

### 6.6. Future Directions

**Figure 5:**
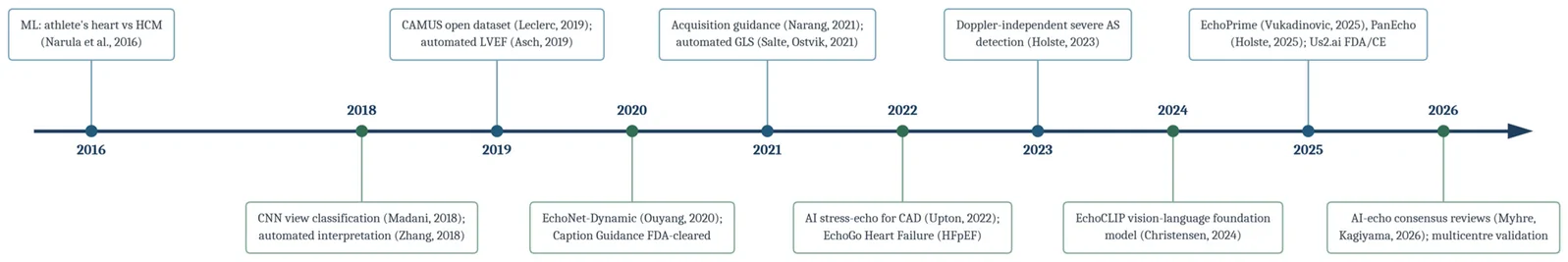
Timeline of selected milestones in AI-enhanced cardiovascular ultrasound (2016–2026), spanning early ML classification, automated segmentation and function, acquisition guidance, regulatory clearances, and the emergence of vision–language and foundation models [10,14,21,22,23,24,27,28,35,36,42,45,46,48,54,75,88].

Several directions are likely to shape the field. Multimodal integration, combining ultrasound with electrocardiographic, laboratory, genomic, and EHR data, will further refine individualized prediction [13,86]. Foundation and multitask models trained across large, heterogeneous datasets point toward general-purpose interpretation rather than narrow tools [32,34,35]. The concept of patient-specific “digital twins” offers a framework for simulating disease progression and treatment response [98], integrating imaging-derived geometry and function with physiological models to test interventions in silico before applying them to the patient; while still early, such approaches could eventually link the reproducible biomarkers AI extracts to individualized, mechanistic prediction. Advances in autonomous acquisition, including robotic systems approaching expert-level autonomous carotid ultrasonography [99], foreshadow examinations performed with minimal human input. Beyond passive interpretation, agentic systems that couple perception with action are emerging: autonomous and robotic acquisition, already demonstrated for carotid ultrasonography [99], may eventually extend to guided or self-driving cardiac scans, while orchestrated pipelines could triage studies, draft structured reports, and flag urgent findings with minimal human input. Continual-learning approaches that safely update models with new data, within predetermined change-control constraints, could keep systems current as practice evolves, provided drift and model collapse are actively monitored [41]. Realizing these possibilities will depend on continued validation, standardization, and thoughtful clinical integration [97]. Figure 5 situates these developments on a decade-long trajectory.

### 6.7. Limitations of This Review

This review has limitations inherent to its narrative design. Although a structured search strategy was applied, study selection was not governed by a formal systematic-review protocol such as PRISMA, and no quantitative meta-analysis was performed; as noted in Section 1, the heterogeneity of study designs, reference standards, and performance metrics precludes direct pooled comparison. We have sought to mitigate the attendant risk of selection bias by prioritizing peer-reviewed, methodologically robust, and clinically representative work and by reporting quantitative performance (including spatial-overlap metrics) wherever available. Finally, the rapidly evolving nature of the field means that some applications, and the regulatory status of specific tools, may advance substantially after publication. We have also deliberately spanned a wide range of applications and maturity levels; while this breadth conveys the scope of the field, it necessarily limits the depth achievable for any single application, each of which could warrant a dedicated systematic review.

## 7. Conclusions

AI-enhanced cardiovascular ultrasound represents one of the most consequential developments in contemporary cardiovascular medicine. By automating acquisition, segmentation, and quantification, AI reduces operator-dependent variability and improves reproducibility and efficiency, gains that are increasingly quantified rather than merely asserted: automated LV segmentation achieves Dice coefficients of approximately 0.92–0.94 on public datasets, automated LVEF is estimated with mean absolute errors of roughly 4–6%, and AI has been shown in test–retest studies to improve the precision of strain measurement relative to conventional analysis [28,33,48,49]. Beyond automation, AI-derived biomarkers, most notably GLS and quantitative plaque burden, extend the diagnostic reach of ultrasound into the subclinical phase, while multimodal predictive models and emerging foundation models translate these biomarkers into individualized risk estimates and general-purpose interpretation.

Important obstacles remain: limited interpretability, uncertain generalizability from public benchmarks to real-world multi-vendor practice, incomplete reproducibility and code/data sharing, and evolving regulatory and ethical frameworks. Prospective randomized evidence is still mixed: although an early blinded trial found AI non-inferior to sonographers for function assessment [88], the first multicenter randomized trial of AI-augmented stress echocardiography did not confirm benefit in an unselected setting [58], tempering enthusiasm and underscoring that diagnostic accuracy and clinical utility are distinct achievements. Concretely, closing these gaps will require prospective, multicenter registries with external validation reported against standardized frameworks; “silent-mode” or shadow deployment of algorithms alongside routine reporting to benchmark real-world performance before autonomous use; integration with EHR and structured-reporting systems; cross-vendor standardization initiatives for measurements such as GLS; privacy-preserving and federated training to build representative datasets; and regulatory adherence to lifecycle-management and predetermined change-control frameworks under FDA GMLP, the EU MDR, and the EU AI Act [10,91,92,93]. With such rigorous validation, standardization, and responsible integration, AI-augmented cardiovascular ultrasound is poised to play a central role in the future of predictive, preventive, and personalized cardiovascular care [1,10].

## Figures and Tables

**Table 1:** Limitations of conventional cardiovascular ultrasound and corresponding AI-enabled solutions.

Limitation of Conventional Ultrasound	Clinical Consequence	How AI Addresses It
Operator-dependent acquisition and interpretation	Inconsistent image quality; variable diagnoses	Automated view recognition and real-time acquisition guidance; standardized analysis
Difficulty obtaining standard views (foreshortening, off-axis/non-standard planes)	Non-diagnostic or geometrically distorted images; systematic measurement bias	Real-time probe-manipulation prompts, automated image-quality scoring, and view-classification that flags foreshortened/off-axis frames
Inter- and intra-observer variability	Reduced reproducibility of measurements	Reproducible, deterministic quantification (e.g., LVEF, GLS)
Geometric assumptions in 2D volume/LVEF estimation	Systematic error; foreshortening	Automated segmentation and 3D/volumetric quantification
Time-consuming manual tracing and measurement	Workflow inefficiency; limited throughput	Rapid automated measurement and reporting
Low sensitivity for subclinical disease	Missed early/preclinical impairment	Detection of subtle patterns and imaging biomarkers
High volume and complexity of imaging data	Information exceeds visual-assessment capacity	Multiparametric data integration and pattern recognition

**Table 2:** Representative AI tasks across the cardiovascular ultrasound workflow.

Workflow Stage/Task	What AI Automates	Clinical Benefit	Representative Study
Acquisition guidance and QC	Real-time probe guidance, image-quality feedback	Diagnostic images by novices; fewer nondiagnostic studies	Narang et al. [14]
View classification	Identification of standard echocardiographic views	Automated study triage and labeling	Madani et al. [27]
Segmentation	Chamber and endocardial border delineation	Geometric basis for reproducible measurement	Leclerc et al. [28]
LVEF quantification	Ejection fraction without manual tracing	Reproducible, rapid functional assessment	Asch et al. [29]; Ouyang et al. [33]
End-to-end interpretation	Automated measurement and reporting	Workflow efficiency; consistency	Zhang et al. [30]; Holste et al. [32]
Disease detection/phenogrouping	Pattern recognition for diagnosis	Decision support; novel patient subgroups	Liu et al. [31]

**Table 3:** Representative publicly available echocardiography datasets used for AI development and benchmarking.

Dataset	Content	Principal Task(s)	Availability
CAMUS [28]	500 patients; 2D apical 2- and 4-chamber with expert tracings	LV/LA segmentation; LVEF	Open
EchoNet-Dynamic [33]	10,030 apical 4-chamber videos with EF labels	LV segmentation; video-based EF	Open (code + data)
EchoNet-LVH [36]	~12,000 parasternal long-axis videos with wall-thickness labels	Hypertrophy phenotyping; HCM/amyloid	Open (code + data)
EchoNet-Pediatric [37]	Pediatric apical/subcostal videos with EF labels	Pediatric LV function	Open
TMED [38]	Labeled 2D views with aortic-stenosis grades	View classification; AS screening	Open
UK Biobank (imaging) [39]	Population cohort with linked imaging and outcomes	Multimodal risk prediction	Managed access

**Table 4:** Representative AI studies for automated left ventricular function quantification, with spatial-overlap and functional-agreement metrics.

Study	Task	Reference Standard	Reported Performance
Leclerc et al. [28]	LV segmentation; EDV/ESV/LVEF (CAMUS)	Expert tracing	Dice ≈ 0.94 (ED)/0.92 (ES); volumes r = 0.95, MAE 9.5 mL; LVEF r = 0.80, MAE 5.6%
Ouyang et al. [33]	Video-based LV segmentation and EF (EchoNet-Dynamic)	Expert tracing	Dice 0.92; LVEF MAE 4.1%; HFrEF AUC 0.97
Asch et al. [29]	Automated LVEF without volume tracing	Expert readers	r = 0.95; bias 1.0%; 95% LOA ± 11.8%; Se 0.90/Sp 0.92 for EF ≤ 35%
Zhang et al. [30]	Fully automated interpretation incl. LVEF	Manual analysis	Automated values within reported inter-observer variability; AUC/Se/Sp not reported
Tsang et al. [17]	Automated 3D LV/LA volumes and LVEF	Manual 3D; CMR	r = 0.87–0.96 (manual); r = 0.84–0.95 (CMR)
Knackstedt et al. [45]	Automated LVEF and GLS	Manual measurement	Feasible in 98% of studies; ICC ≈ 0.83–0.84; ~8 s per study; automated EF/LS without measurement variability
Ghorbani et al. [44]	DL interpretation; structures and function	Expert annotation	Expert-level across multiple tasks; comparable AUC/R^2^ not reported in a uniform metric

**Table 5:** Representative AI studies for myocardial strain and GLS.

Study	Focus/Design	Reference Standard	Key Finding
Salte et al. (2021) [46]	Automatic LV strain (GLS)	Reference STE	Bias −0.7%; 95% LOA −4.6 to 3.1%; feasibility 93%
Ostvik et al. (2021) [47]	DL myocardial function imaging	Reference STE	Automated motion/strain estimation feasible; quantitative agreement metrics not reported
Salte et al. (2023) [48]	Test–retest precision	Manual STE	Improved test–retest precision vs. manual STE; numerical LOA not reported
Nyberg et al. (2024) [49]	Regional strain	Manual STE	Improved regional-strain test–retest reproducibility; ICC not reported
Rogstadkjernet et al. (2024) [50]	Repeatability and accuracy	Experienced readers	Mean absolute difference 0.75 GLS units (95% CI 0.58–0.92) vs. experienced readers
Sveric et al. (2024) [51]	GLS standardization	Multi-reader	R = 0.92; bias 0.7%; 95% LOA −3.5 to 4.8%; ICC 1.0; feasibility 89%
Stowell et al. (2024) [52]	GLS from open UK-wide data	Human readers	Median absolute error 1.3 GLS units; r = 0.91 vs. multi-expert consensus
Kuwahara et al. (2024) [54]	Strain for CTRCD surveillance	Conventional strain	Reproducible subclinical CTRCD detection; comparative AUC not reported

**Table 6:** Representative AI studies for carotid and vascular ultrasound.

Study	Task	Approach	Reported Performance/Outcome
Araki et al. [66]	Stroke risk stratification from plaque morphology	ML	Validated stroke-risk stratification; AUC/Se/Sp not reported in a comparable metric
Sharma et al. [67]	Plaque tissue characterization (framework)	ML	Feasibility of automated plaque tissue characterization; quantitative metrics not reported
Saba et al. [68]	Internal carotid plaque characterization	DL	High classification accuracy; specific AUC/Se/Sp not reported here
Skandha et al. [69]	3D plaque classification (Atheromatic 2.0)	DL	Improved automated risk stratification; comparative AUC not reported
Zhou et al. [70]	Plaque segmentation/TPA	DL (modified U-Net)	r ≈ 0.99 vs. manual; ~8 ms per plaque
Zhang & Zhao [71]	Plaque detection and classification	DL	Accurate plaque detection and classification; AUC/Se/Sp not reported here
Jamthikar et al. [73]	Office-based cardiovascular/stroke risk	ML	Low-cost office-based risk stratification; comparative AUC not reported

**Table 7:** Representative AI cardiovascular-ultrasound tools with regulatory clearance (status as of 2026; illustrative, not exhaustive). Regulatory status was verified against the FDA AI/ML-Enabled Medical Devices list and EU databases and should be re-checked at the time of reading, as approvals evolve rapidly [87].

Tool	Developer	Principal Function	Regulatory Status
Caption Guidance	Caption Health (GE HealthCare)	Real-time AI acquisition guidance and quality control for novices	FDA De Novo (2020)
Us2.ai (AI Echo Copilot)	Us2.ai	Fully automated, vendor-agnostic LVEF, GLS, diastolic function, AS grading; single-view amyloidosis detection	FDA-cleared; CE-marked
EchoGo Core/Pro	Ultromics	Automated quantification/strain (Core); CAD decision support (Pro)	FDA 510(k); CE-marked
EchoGo Heart Failure	Ultromics	HFpEF detection from a single echocardiographic view	FDA-cleared (breakthrough device)
EchoGo Amyloidosis	Ultromics	Cardiac-amyloidosis screening aid	FDA-cleared (TAP pathway, 2024)
LVivo	DiA Imaging Analysis	Automated LV function/EF and cardiac quantification	FDA-cleared; CE-marked

**Table 8:** Maturity and evidence level of AI applications in cardiovascular ultrasound.

Application	Maturity	Evidence Level	Remaining Steps to Routine Use
View classification and segmentation	High	Strong; multiple validated models	Workflow integration; vendor-neutral deployment
LVEF/volume quantification	High	Strong; positive blinded RCT vs. sonographers [88]	Prospective outcome validation
Strain/GLS	Moderate–high	Strong reproducibility data	Cross-vendor standardization
Stress echo/CAD detection	Moderate	Multicenter RCT (PROTEUS; mixed result)	Trials with clinical-outcome endpoints
Valvular (aortic stenosis) detection	Moderate	Emerging; cleared tools available	Prospective screening trials
Cardiomyopathy detection (HCM, amyloidosis)	Moderate	Strong external validation	Prospective screening; outcome linkage
Diastolic function/Doppler interpretation	Moderate	Multicohort validation	Guideline concordance at scale
Carotid plaque/stroke risk	Moderate	Substantial methodological work	Outcome-linked validation
Fetal/congenital screening	Moderate	High accuracy in test sets	Prospective, real-world screening
HFpEF phenogrouping/risk prediction	Investigational	Proof-of-concept	Prospective, multimodal validation
Therapy personalization	Investigational	Early	Randomized confirmation

## Data Availability

No new data were created or analyzed in this study. Data sharing is not applicable to this article.

## Abbreviations

The following abbreviations are used in this manuscript:

2D/3D	Two-/three-dimensional
AI	Artificial intelligence
AS	Aortic stenosis
AUC	Area under the ROC curve
CAD	Coronary artery disease
CE	Conformité Européenne
CHD	Congenital heart disease
CMR	Cardiac magnetic resonance
CNN	Convolutional neural network
CRT	Cardiac resynchronization therapy
CTRCD	Cancer therapy-related cardiac dysfunction
CVD	Cardiovascular disease
DL	Deep learning
GAN	Generative adversarial network
RCT	Randomized controlled trial
RV	Right ventricle/right ventricular
DSC	Dice similarity coefficient
EDV/ESV	End-diastolic/end-systolic volume
EHR	Electronic health record
EU MDR	European Union Medical Device Regulation
FDA	U.S. Food and Drug Administration
GLS	Global longitudinal strain
GMLP	Good Machine Learning Practice
HCM	Hypertrophic cardiomyopathy
HF	Heart failure
HFpEF	Heart failure with preserved ejection fraction
LA	Left atrium/left atrial
LLM	Large language model
LV	Left ventricle/left ventricular
LVEF	Left ventricular ejection fraction
MAE	Mean absolute error
ML	Machine learning
POCUS	Point-of-care ultrasound
QC	Quality control
RWMA	Regional wall motion abnormality
STE	Speckle-tracking echocardiography
TDI	Tissue Doppler imaging
TPA	Total plaque area
VHD	Valvular heart disease
ViT	Vision transformer
VLM	Vision–language model
